# Multi-Level Effects of Acute Heat Stress on Gill Tissue of *Gymnocypris eckloni*: Integrating Histopathology, Biochemistry, Apoptosis and Transcriptomics

**DOI:** 10.3390/ani16121762

**Published:** 2026-06-08

**Authors:** Yanzhen Dong, Zhiqiang Zhang, Changlun Xiao, Dayong Xu, Sihong Deng, Pan Shang, Mingkun Luo, Ying Wang

**Affiliations:** 1 Key Laboratory of Application of Ecology and Environmental Protection in Plateau Wetland of Sichuan, Xichang University, Xichang 615013, China; xcc04000019@xcc.edu.cn (Y.D.); zhiqiangzhang0908@163.com (Z.Z.); xcc04000017@xcc.edu.cn (D.X.); 13795662066@126.com (S.D.); shpan1017@126.com (P.S.); 2 Institute of Animal Husbandry and Fisheries, Yibin Academy of Agricultural Sciences, Yibin 644600, China; changlunxiao@163.com; 3 Key Laboratory of Freshwater Fisheries and Germplasm Resources Utilization, Freshwater Fisheries Research Center, Chinese Academy of Fishery Sciences, Wuxi 214128, China; luomingkun@ffrc.cn

**Keywords:** *Gymnocypris eckloni*, acute heat stress, gill, histopathology, transcriptome

## Abstract

Extreme high-temperature events driven by global climate change are occurring with increasing frequency, posing a serious threat to the stability of aquatic ecosystems. The Tibetan schizothoracin (*Gymnocypris eckloni*), a cold-water fish species endemic to the Qinghai–Tibet Plateau, is highly sensitive to temperature fluctuations and serves as an ideal model for studying the effects of climate change on fish. As a key organ for fish to perceive environmental changes, the gills’ comprehensive response mechanism has not yet been fully elucidated. This study investigated the effects of acute heat stress on the gill tissue of *G. eckloni*. The results showed that acute heat stress caused severe histopathological damage in the gills, including lamellar curling, epithelial cell detachment, and edema, with a significant increase in apoptosis. Biochemical analysis revealed elevated levels of cortisol, glucose, and ATPase activity in serum, as well as increased MDA content and CAT activity in the gills. Transcriptomic analysis identified 2304 DEGs. Upregulated DEGs were significantly enriched in pathways related to inflammatory response, TNF signaling, ferroptosis, and apoptosis, while downregulated DEGs were primarily involved in peroxisome metabolism, cell cycle, and steroid biosynthesis. This study confirms that acute heat stress induces structural damage and functional impairment in the gills by activating inflammatory and apoptotic pathways and disrupting redox homeostasis. It elucidates the immediate molecular and physiological responses of *G. eckloni* gills to acute heat stress. Follow-up experiments will be conducted at multiple time points, across different temperature gradients, and under chronic stress conditions to gain a more comprehensive understanding of the adaptive potential of high-altitude fish to climate warming, thereby providing a scientific basis for the development of conservation strategies.

## Simple Summary

Global warming brings more frequent extreme high temperatures, which harms aquatic life. We studied a unique cold-water fish living on the Qinghai–Tibet Plateau, as it is easily affected by temperature changes. We explored how sudden high temperatures impact the gills of this fish, a key body part that senses the surrounding environment. Our findings show extreme heat damages gill structure and disturbs normal physical functions. It also triggers inflammation and cell death in gill tissues. This work reveals how this plateau fish copes with sudden heat. The results help us learn about fish adaptation to a warmer climate and support practical efforts to protect local aquatic animal resources.

## 1. Introduction

Extreme high-temperature events caused by global climate change are occurring more frequently and have become a key environmental factor threatening the stability of aquatic ecosystems [1]. As ectothermic animals, fish have their physiological functions and survival directly regulated by environmental temperature. Heat stress triggers a series of stress responses ranging from the molecular to the organ level, and in severe cases, can lead to population decline and biodiversity loss [2]. For example, cold-water fish such as salmon and trout show a significant decline in survival under elevated temperatures when the habitat temperature approaches their physiological tolerance threshold [3]. Under high-temperature stress, the activities of SOD, CAT, and cortisol in the blood of American shad (*Alosa sapidissima*) are significantly elevated, and the levels of antioxidant and heat shock protein family genes or proteins are markedly upregulated [4]. Furthermore, climate change may substantially reduce the habitat suitability of specific fish species, leading to population declines and increased extinction risk [5]. Water temperature is also closely related to the reproductive success of fish; temperature changes may cause reproductive failure, thereby threatening the long-term sustainability of fish populations [6]. Therefore, understanding the impacts of climate warming on fish survival and elucidating the physiological and molecular response mechanisms of fish to high-temperature stress are of great theoretical and practical significance for assessing the effects of climate change on aquatic organisms, predicting shifts in species distribution, and formulating conservation strategies.

The Tibetan schizothoracin (*Gymnocypris eckloni*) is an important cyprinid fish species in the subfamily Schizothoracinae. It is primarily found in rivers, lakes, and other water bodies on the Qinghai–Tibet Plateau and in the upper reaches of the Yellow River. As a species endemic to and adapted for the cold-water plateau environment, it plays a significant role in maintaining the ecological balance of plateau aquatic ecosystems. Having long inhabited the cold, oligotrophic, and variable plateau environment, it is highly sensitive to environmental stressors such as temperature fluctuations, making it an ideal model for studying the physiological and ecological impacts of climate change on plateau fish [7]. Since the successful artificial breeding of *G. eckloni* in Qinghai and Sichuan provinces, China, in 2008, it has become a commercially valuable species and is widely farmed in southwestern China [8]. The optimal temperature for its growth and reproduction is 16–18 °C, and growth rates decline if temperatures fall outside this range [9]. In addition, studies have found that when adapting to highly saline–alkaline environments, the gills and kidneys of the Qinghai Lake naked carp (*G. przewalskii*) undergo plastic morphological changes and activate the expression of genes related to ion transport, cell junctions, and metabolic regulation [10]. Whole-genome studies have also revealed the genetic basis for adaptation to extreme environments, such as positive selection or specific expansion in gene families associated with osmoregulation, including aquaporin (*aqp*3) and ion transporter genes [11]. In coping with oxidative stress, the accumulation of ROS under high-temperature stress triggers a response from the antioxidant enzyme system in *G. eckloni*, such as the upregulation of oxidative stress-related genes observed in its gill and liver tissues under copper ion stress [12]. As the metabolic hub of the organism, the liver undergoes significant metabolic reprogramming under thermal stress, involving extensive adjustments to pathways related to glucose, lipid, and amino acid metabolism [13]. Research by Zhou et al. [14] on *G. eckloni* indicates that heat stress leads to liver tissue damage, lipid deposition in hepatocytes, mitochondrial structural abnormalities, and the induction of apoptosis. Biochemical analysis showed that oxidative stress activated antioxidant enzymes such as CAT, while the organism’s energy demand increased. Omics analysis further revealed that the protein processing pathway in the endoplasmic reticulum was significantly activated, with upregulation of key genes related to the unfolded protein response (such as *hsp40*, *hsp70*, *ero1l* and *pdia4*) to help clear misfolded proteins and maintain cellular homeostasis. These studies collectively demonstrate that high-temperature exposure profoundly affects the physiological state and metabolic functions of *G. eckloni,* leading to oxidative damage and energy metabolism imbalance. In-depth analysis of its response mechanisms to high-temperature stress is crucial for understanding the environmental adaptability of plateau fish and developing targeted conservation strategies.

Gill tissue is a key organ in fish for gas exchange, osmoregulation, and waste excretion, serving as the primary interface directly exposed to and for sensing environmental pressures. It responds rapidly to environmental stressors such as temperature fluctuations and is often used as an indicator for assessing fish health and adaptability [15]. High-temperature stress can cause pathological changes in the morphological structure of fish gills, including epithelial hyperplasia, tissue damage, increased apoptosis, and alterations in gene expression regulation. These physiological and molecular responses may ultimately affect individual growth, reproduction, and even population stability [16]. Although such studies have been reported in various fish species, a comprehensive analysis integrating enzyme activity, morphology, apoptosis, and gene expression from multiple perspectives remains to be explored in *G. eckloni*. Therefore, this study aims to systematically investigate the multi-level effects of high-temperature stress on the gill tissue of *G. eckloni*, elucidating the patterns of enzymatic response, morphological changes, apoptotic dynamics, and key gene regulatory networks under thermal stress. The findings are expected to provide new insights into the adaptive evolution of plateau fish to environmental stress and emphasize the necessity of strengthening species conservation in the context of global warming.

## 2. Materials and Methods

### 2.1. Ethics Approval

In this study, the protocol for animal sample collection strictly complies with relevant Chinese laws and regulations. All experimental procedures involving animals were conducted in accordance with the ethical standards formally approved by the Animal Ethics Committee of Xichang University (Approval No.: xcc2024017) on 18 January 2024.

### 2.2. Experiment Animals and Heat Stress Treatment

*G. eckloni* weighing 150 ± 8 g were purchased from Xide Zhengyuan Aquatic Co., Ltd. (Xichang, Sichuan, China) and transported to the laboratory of the College of Animal Science and Technology, Xichang University. The fish were randomly divided into two groups, with three replicate tanks for each group, each containing 10 fish. The fish were reared in rectangular glass tanks (80 cm × 60 cm × 60 cm) equipped with electric heaters and thermometers. An HWS-type constant temperature and humidity incubator (HWS-1000, Ningbo, China) maintained the water temperature at 13 °C. Throughout the experiment, the fish were fed three times daily, and the water pH was kept at 6.6~6.9, with dissolved oxygen concentrations above 5 mg/L. After a one-week acclimatization period, the following temperature stress treatment groups were established: Control Group (CG), with water temperature maintained at 13.0 ± 0.5 °C; and Acute Heat Stress Group (AH), in which, following the acute heat stress protocol for *G. eckloni* by Zhou et al. [14] and referencing the protocols by Dagoudo et al. [17] and Liu et al. [18], the water temperature was increased by 1 °C per hour until reaching 28.0 ± 0.5 °C, then maintained at that temperature for 12 h. No fish mortality occurred during the acute stress experiment. Initial symptoms included accelerated swimming and rapid breathing; in the middle and late stages, swimming imbalance was observed. As stress intensified, feeding activity decreased significantly.

### 2.3. Sample Collection

Nine fish were randomly selected from the AH group and the CG group (three fish per tank), respectively, and anesthetized with 30 mg/L MS-222 (Sigma, St. Louis, MO, USA). Blood was collected using a 1 mL disposable syringe, followed by collection of gill tissue for later use. Three gill tissue samples were obtained: the first subsample was fixed in 4% paraformaldehyde solution (Shanghai Shengong Bioengineering Co., Ltd., Shanghai, China) for histopathological analysis and apoptosis detection (three fish per group); the second and third subsamples were rapidly frozen in liquid nitrogen and stored at −80 °C for transcriptomic analysis (three fish per group) and quantitative reverse transcription polymerase chain reaction (qRT-PCR) analysis (three fish per group), respectively.

### 2.4. Histopathology and Apoptosis Analysis

The gill tissues were fixed in paraformaldehyde for 24 h, dehydrated through a series of ethanol gradients, cleared with xylene, embedded in paraffin, and sectioned into 4–5 μm thick slices. The sections were stained with hematoxylin and eosin (Solarbio, Beijing, China) and mounted with neutral balsam. Morphological observation was performed using a light microscope (Zeiss, Oberkochen, Germany) to evaluate pathological changes in the gill tissue.

Apoptotic cells in the gills were detected using a TUNEL apoptosis detection kit (Solarbio, Beijing, China). After dewaxing and rehydration through an ethanol gradient, tissue sections were treated with proteinase K at 37 °C for 30 min. They were then incubated at 37 °C for 2 h with a labeling solution containing terminal deoxynucleotidyl transferase, buffer, and fluorescein-dUTP, followed by DAPI counterstaining. The sections were examined under a fluorescence microscope (Eclipse 50i, Nikon, Tokyo, Japan), and ImageJ (Version2.0.0) image analysis software (developed by the National Institutes of Health, Bethesda, MD, USA) was used for quantitative analysis of cell viability in the gill tissue.

### 2.5. Biochemical Index Detection

Blood samples from the AH and CG were stored at 4 °C for 12 h, then centrifuged at 5000× *g* rpm for 10 min. The supernatant from the total serum was collected for the measurement of ATPase, T-AOC, Glu and cortisol. Concurrently, the frozen gill tissue samples were used for oxidative stress analysis, including the detection of MDA, SOD, CAT and LDH.

### 2.6. Transcriptome Sequencing

Total RNA was extracted from gill tissue (*n* = 3 per group) using TRIzol^®^ Reagent (CWBio, Suzhou, China) according to the manufacturer’s instructions. RNA concentration was determined by agarose gel electrophoresis, and RNA integrity was assessed with an Agilent 2100 Bioanalyzer (Agilent Technologies, Santa Clara, CA, USA). mRNA was then enriched using the NEBNext Ultra™ RNA Library Prep Kit for Illumina (NEB, Ipswich, MA, USA), and reverse transcription was performed to generate cDNA libraries. After purification, the cDNA libraries were sequenced on an Illumina NovaSeq 6000 platform (2 × 150 bp) by Shanghai OE Biotech Co., Ltd. (Shanghai, China). Raw reads were filtered with fastp (v0.18.0) to obtain high-quality clean reads. Paired-end sequences were aligned to the reference genome of *G*. *eckloni* (GenBank accession: GCA_027564155.1) [19] using HISAT2 (v2.1.0). The Q30 base coverage ranged from 93.98% to 94.54%, with an average GC content of 46.68%. After alignment, protein-coding genes with zero counts across all samples were removed. Gene expression levels were annotated and analyzed using the FPKM method, and differential expression analysis between the two groups was performed using the DESeq R package (v1.18.0). Genes with a false discovery rate (FDR) < 0.05 and an absolute fold change ≥ 2 were defined as DEGs. The significance of these differences was tested using the negative binomial distribution test.

All DEGs were then mapped to the Gene Ontology (GO) database (http://www.geneontology.org/, accessed on 10 April 2025) and the Kyoto Encyclopedia of Genes and Genomes (KEGG) database (http://www.genome.jp/kegg/, accessed on 22 April 2025) using the clusterProfiler R package. The number of DEGs in each GO term was counted, and the significance of enrichment in each GO term was calculated using the hypergeometric distribution algorithm. Pathway analysis of DEGs was performed using the KEGG database and annotation results, and the significance of enrichment in each pathway was calculated using the hypergeometric distribution test. The raw transcriptome sequencing data have been deposited in the NCBI database under accession number PRJNA1153723.

### 2.7. Quantitative Real-Time PCR Analysis

To validate the accuracy of the transcriptome sequencing data, we randomly selected 10 DEGs for validation using qRT-PCR. The selected genes and their corresponding PCR primers are listed in Table S5. The specific qPCR protocol followed the experimental methods previously described by Luo et al. [20]. All samples were analyzed with three technical replicates and three biological replicates. *β*-actin was used as the internal reference, and the relative RNA expression levels of the target genes were calculated using the 2^−ΔΔCt^ method [21].

### 2.8. Statistical Analysis

Since the heat stress treatment was conducted at the aquarium level, with each aquarium serving as an independent experimental unit, we addressed potential pseudo-replication by using the aquarium-level mean as the unit of analysis. For each aquarium, measurements from three fish were averaged to obtain a single value per aquarium (*n* = 3 aquariums per group). These tank-level means were then used for statistical comparisons. Data analysis was performed using SPSS software (v25.0) (SPSS Inc., Chicago, IL, USA). All experimental data are presented as mean ± standard deviation. A *t*-test was used to determine whether differences between the control and experimental groups were statistically significant. Statistical significance is indicated as follows: * *p*< 0.05; ** *p*< 0.01; *** *p*< 0.001. Non-significant differences are denoted as “ns” (not significant). Figures were generated using GraphPad Prism software (v9.0).

## 3. Results

### 3.1. Effect of Heat Stress on Gill Histopathology and Apoptosis

**Figure 1:**
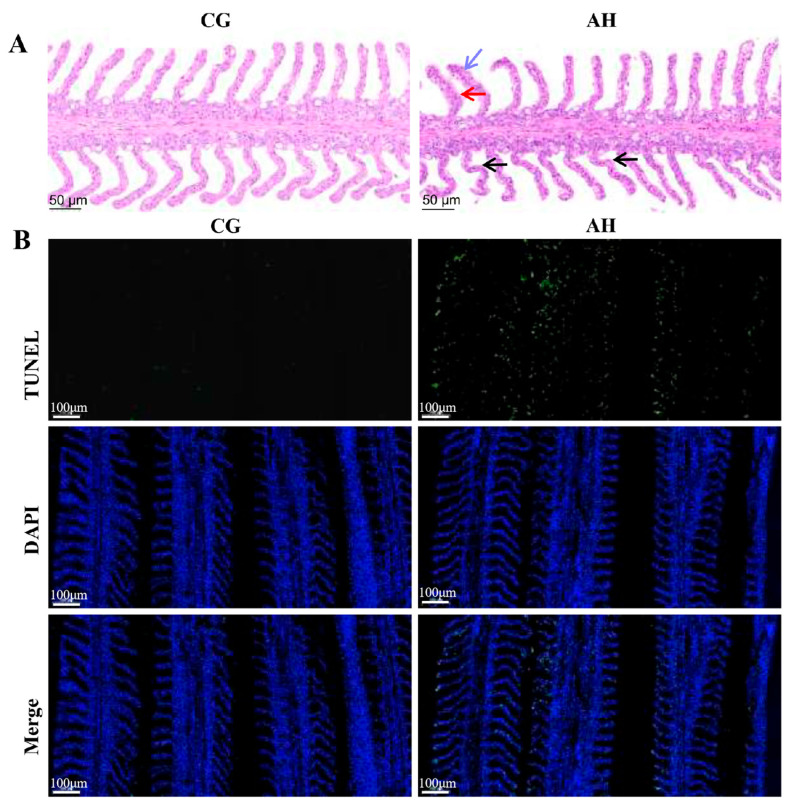
Heat stress induces histopathological changes and cell apoptosis in fish gill tissues. (**A**) HE-stained gill tissue, scale bar = 50 μm. Curled secondary gill filaments (black solid arrows), epithelial cell detachment (red solid arrows), and edema of secondary gill filaments (blue solid arrows). (**B**) TUNEL staining of gill tissue, scale bar = 100 μm. Cells exhibiting green fluorescence indicate apoptotic cells.

We found that acute heat stress caused severe pathological damage and significant apoptosis in the gill tissues of *G. eckloni* (Figure 1). The gill tissue of the control group (CG) displayed intact, neatly arranged gill lamellae with an unbroken epithelium, overall clear cellular structure, and normal morphology. In contrast, the gill tissue of the heat-stressed group (AH) exhibited varying degrees of histological changes, specifically including curled gill lamellae, epithelial cell sloughing, gill lamellae edema, vacuolar degeneration, blurred cell morphology, and nuclear lysis (Figure 1A). The results of the apoptosis assay showed no obvious green fluorescence signal (indicating apoptotic cells) in the gill tissues of the control group. In contrast, green fluorescence was widely distributed in the secondary gill filaments of the heat-stressed group, indicating a higher number of TUNEL-positive cells. This suggests that apoptosis was significantly activated following heat stress, and the damage was more severe (Figure 1B).

### 3.2. The Effects of Heat Stress on Biochemical Indicators

**Figure 2:**
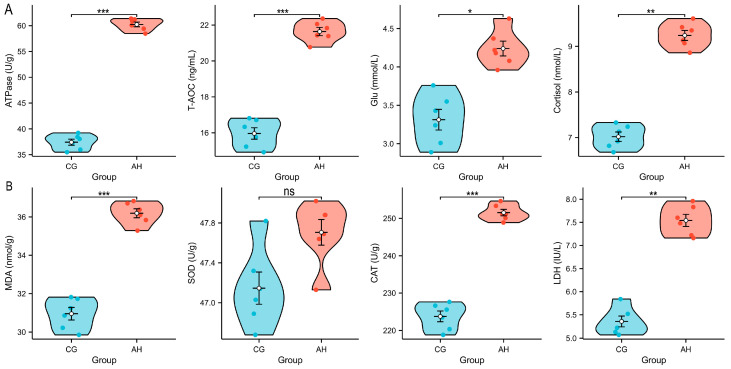
Effects of acute heat stress on physiological and biochemical parameters. (**A**) in the top row shows changes in serum biochemical parameters; (**B**) in the bottom row shows changes in oxidative stress markers in gill tissue. * *p* < 0.05; ** *p* < 0.01; *** *p* < 0.001.

As shown in Figure 2, changes in relevant physiological and biochemical indicators in the serum and gill tissues of *G. eckloni* under acute heat stress are illustrated. The levels of total ATPase and total T-AOC in the serum of the AH were significantly higher than those in the control group (*p* < 0.001). Blood glucose levels in the AH were also significantly elevated compared with the CG (*p* < 0.05). Additionally, cortisol levels in the AH were significantly increased (*p* < 0.01) (Figure 2A). For oxidative stress indicators in gill tissue (Figure 2B), MDA and CAT levels in the AH were significantly higher than those in the control group (*p* < 0.001). LDH activity was significantly increased under acute heat stress (*p* < 0.05), while SOD activity did not show significant changes after heat stress.

### 3.3. Transcriptome Analysis of the Gills of the G. eckloni Under Acute Heat Stress

To investigate the extent of genetic changes associated with the acute heat stress response, we performed RNA sequencing on gill tissue samples from the CG and AH. The raw read depth ranged from approximately 41.9 to 49.83 Mb (Table 1). After quality control based on the Q30 score, each sample yielded 39.43 million to 47.03 million high-quality reads, representing 93.98% to 94.54% of the total, indicating sufficient sequencing depth (Table 1). All raw sequences have been submitted to the NCBI database under accession number PRJNA1153723.

**Figure 3:**
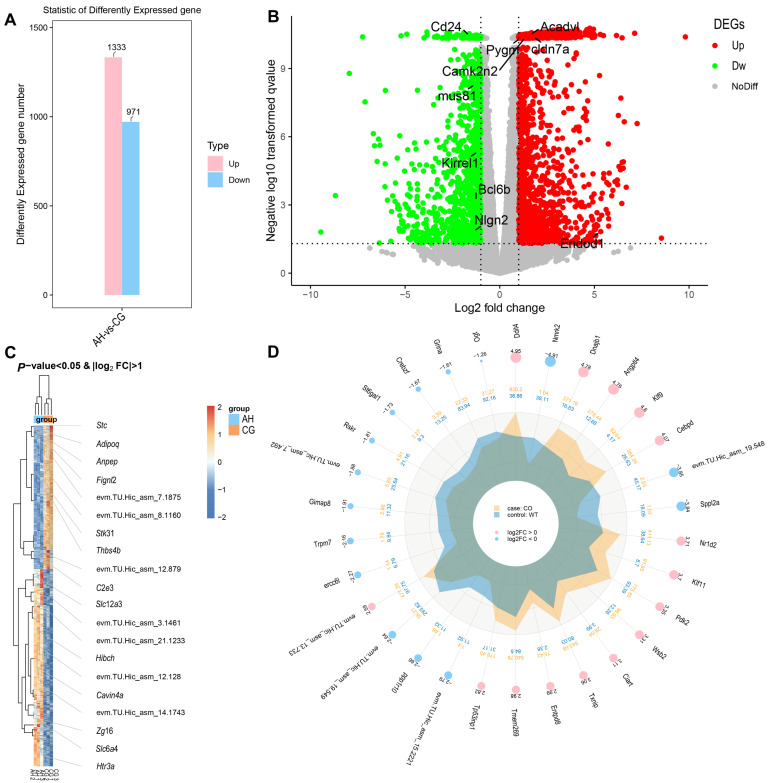
Analysis of DEGs. (**A**) Statistical bar chart of DEGs, where “Up” indicates the number of upregulated genes and “Down” indicates the number of downregulated genes. (**B**) Volcano plot of differentially expressed genes, with gray dots representing non-significant genes, and red and green dots representing significant DEGs. (**C**) Hierarchical clustering heatmap of DEGs. (**D**) Radar plot of DEGs: the innermost (first) ring displays upregulated (light red) and downregulated (light blue) genes, with the size of each segment varying according to the |log_2_(FC)| value. The middle (second) ring represents the average expression level in the experimental group (outer data) and the control group (inner data). The outermost (third) ring shows the average expression level of each individual gene in both the experimental and control groups.

We used HISAT2 software (Version 2.0.4) to align the clean reads to the *G. eckloni* reference genome, obtaining genomic information for these sequences and sequence characteristics specific to each sequencing sample. The results showed that the proportion of uniquely mapped reads ranged from 70.54% to 72.01% (Table S1). Between 19,951 and 22,207 protein-coding genes were identified across the six samples (Figure S1A). Box plots of FPKM values for gene expression across samples indicated a relatively uniform data distribution and good sequencing quality (Figure S1B). The inter-sample correlation coefficients based on gene expression levels were all higher than 0.94. Cluster analysis clearly distinguished the acute heat stress treatment group from the control group, further demonstrating the strong correlation among samples (Figure S1C,D). This suggests that three replicates per treatment group are sufficient for the next step of analysis. Analysis of DEGs identified a total of 2304 DEGs (*q*-value < 0.05 & |log_2_FC| > 1.0) between the AH and the CG, with 1333 upregulated and 971 downregulated (Figure 3A & Table S2). Volcano plots visually demonstrated that genes such as *pygm* and *camk2n2* were significantly upregulated in the AH, while genes such as *nlgn2* and *bcl6b* were significantly upregulated in the CG (Figure 3B). Hierarchical clustering analysis of DEGs revealed distinct transcriptional profiles between the AH and CG, indicating significant gene expression reprogramming under heat stress conditions (Figure 3C). Radar plots of DEGs further visualized 15 significantly upregulated genes (e.g., *Ddit4*, *Klf9*, *Nmrk2* and *Sppl2a*) shared between the two groups (Figure 3D).

**Figure 4:**
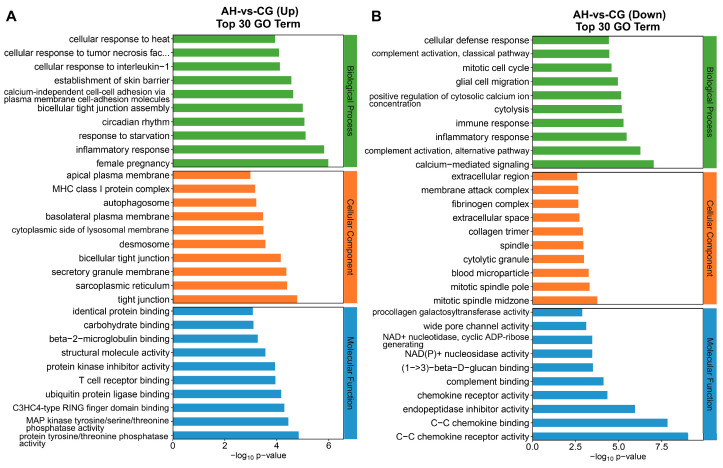
GO enrichment analysis results. (**A**) Bar plot showing the top 30 enriched GO terms among upregulated genes, and (**B**) bar plot showing the top 30 enriched GO terms among downregulated genes. In the plots, the vertical axis represents GO term names, and the horizontal axis represents –log_10_ (*p*-value).

Analysis of GO terms for all DEGs showed that these genes were primarily enriched in the “Inflammatory Response” and “Immune Response” categories within the “Biological Processes” category (Figure S2 & Table S3). For “cellular components”, enrichment was mainly found in terms such as “extracellular space” and “extracellular region,” while for “molecular functions”, it was mainly observed in terms such as “C-C chemokine” and “MAP kinase tyrosine/serine/threonine phosphatase activity” (Figure S2 & Table S3). Among the significantly upregulated genes, we observed significant enrichment in inflammatory response, tight junction, and protein tyrosine/threonine phosphatase activity (Figure 4A). In contrast, GO terms associated with significantly downregulated genes were primarily enriched for calcium-mediated signaling, cytolytic granule, and endopeptidase inhibitor activity (Figure 4B). These changes in GO functions may be related to the molecular events underlying heat stress-induced gill tissue pathology. Changes in gene expression levels lead to structural damage, such as gill filament curling, epithelial detachment, and edema, and may result in apoptosis in gill tissue by affecting the transcriptional regulation of cell survival and immune response pathways.

**Figure 5:**
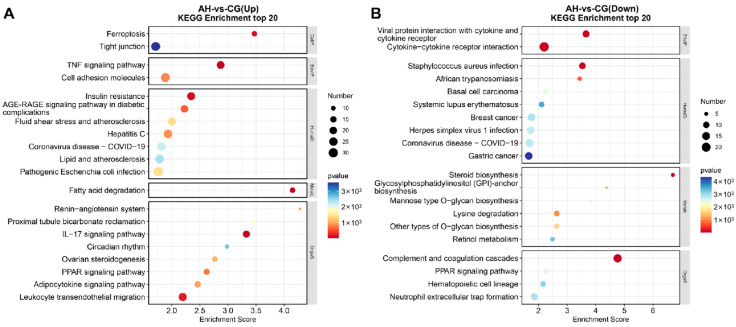
KEGG pathway enrichment analysis results. (**A**) Top 20 bubble plot of KEGG pathway enrichment for upregulated genes; (**B**) Top 20 bubble plot of KEGG pathway enrichment for downregulated genes. The horizontal axis represents the enrichment score. Larger bubbles indicate pathways with a greater number of differentially expressed protein-coding genes. The bubble color transitions from blue to white to yellow to red, with a lower *p*-value corresponding to a redder color and thus higher statistical significance.

To further elucidate the molecular signaling pathway changes underlying gill pathology and apoptosis induced by acute heat stress, we performed KEGG pathway classification and enrichment analysis on the upregulated and downregulated genes in the AH and CG groups (Figure 5 & Table S4). Enrichment analysis of the top 20 KEGG pathways associated with upregulated genes identified key signaling pathways with high scores and statistical significance, including significantly upregulated ferroptosis, TNF signaling pathway, and cell adhesion molecules (Figure 5 and Figure S3). In addition, pathways related to apoptosis and tissue damage, such as the IL-17 signaling pathway and PPAR signaling pathways, were also significantly enriched (Figure 5A). In the analysis of downregulated genes, we performed functional analysis of the top 20 KEGG-enriched pathways (Figure 5B). We observed significant enrichment of pathways related to cellular processes, including cytokine–cytokine receptor interaction. Among metabolic pathways, steroid biosynthesis showed a high enrichment score and the most significant *p*-value among all pathways analyzed. Glycosylphosphatidylinositol (GPI)-anchor biosynthesis was also highly enriched. These results suggest that gene downregulation may affect lipid metabolic processes, such as steroid synthesis, and that steroids play a crucial role in cell membrane structure and cell signaling.

### 3.4. qRT-PCR Verification

**Figure 6:**
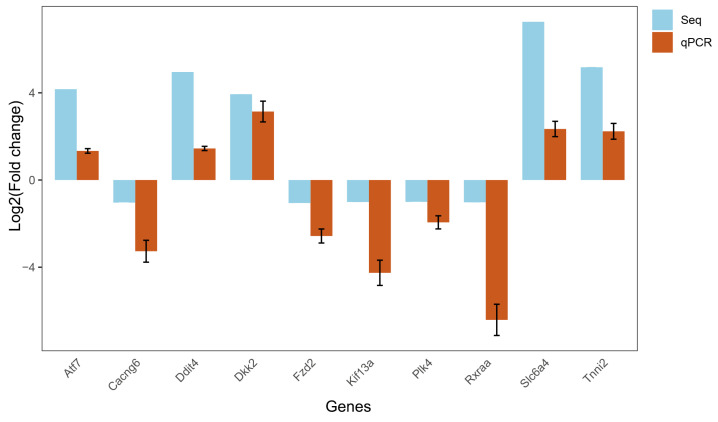
qRT-PCR verification of the expression profiles in AH-vs-CG comparison group.

To further validate gene expression levels using RNA-Seq, we randomly selected 10 genes from the comparison group for qPCR validation (Table 2). Detailed information on these genes, including 5 upregulated and 5 downregulated genes, is shown in Figure 6. The qPCR results indicate that the expression patterns of these 10 genes are consistent with the RNA-Seq results, demonstrating the reliability and accuracy of the transcriptomic data.

## 4. Discussion

Current research on *G. eckloni* has primarily focused on elucidating the physiological and molecular mechanisms underlying its adaptation to high-salinity and alkaline environments, including osmotic regulation, ion transport, ammonia excretion, and gut microbiota [22,23,24]. In contrast, studies on the molecular response mechanisms of *G. eckloni* to the increasingly severe stress of high-temperature exposure remain limited. Through histopathological, physiological, biochemical, and transcriptomic analysis, this study systematically revealed the multifaceted damage caused by acute heat stress to the gill tissue of *G. eckloni* and its underlying molecular regulatory mechanisms. Histopathological observations and TUNEL assay results confirmed that acute heat stress caused severe damage to the gill structure, resulting in characteristic lesions such as curled gill filaments, epithelial cell sloughing, edema, and vacuolar degeneration, accompanied by widespread apoptosis. This phenotype is consistent with the responses of many other fish species to heat stress [25,26], indicating that the gills, as the primary respiratory and osmotic regulatory organs in direct contact with the environment, are extremely sensitive to temperature stress. Damage to gill structure inevitably impairs key physiological functions such as gas exchange and ion regulation.

Physiological and biochemical data show that total ATPase activity and T-AOC in serum increased significantly after heat stress, suggesting that the body may have compensatorily activated energy metabolism and antioxidant defense mechanisms in response to the high-temperature environment. At the same time, serum glucose and cortisol levels also increased significantly, which are typical signals of a stress response and classic indicators of hypothalamic–pituitary–adrenal (HPA) axis activation in fish under stress, indicating that the organism is experiencing high levels of physiological stress [27]. In gill tissue, MDA content and CAT activity increased significantly, and LDH activity was also elevated, indicating that heat stress induced a strong oxidative stress response [28]. Excessive production of ROS may lead to lipid peroxidation, directly damaging the cell membrane systems, which is consistent with histopathological observations of vacuolar degeneration and membrane structural disruption in gill tissue [29]. In summary, the results of biochemical parameters indicate that under heat stress conditions, the organism’s endocrine function, energy metabolism, and antioxidant defense capacity all undergo significant changes.

Transcriptomic analysis further revealed the molecular regulatory mechanisms underlying the pathological and biochemical changes in gill tissue. The study identified 2304 DEGs, among which the expression levels of immune-related genes such as Toll-like receptors (*TLRs*), interleukin-1*β* (*IL*-1*β*), and tumor necrosis factor-α (*TNF*-α) showed significant changes, indicating that acute heat stress induced alterations in immune-related genes in gill tissue [30]. GO functional enrichment analysis showed that upregulated genes were significantly enriched in terms such as “inflammatory response”, “T-cell receptor binding”, and “secretory granule membrane”, while downregulated genes were associated with functions such as “immune response” and “calcium-mediated signaling”. This suggests that the organism initiated a defense or damage response centered on inflammatory and immune reactions, while certain key cellular signaling processes were also altered to adapt to changes in the external environment [31]. Furthermore, KEGG pathway enrichment analysis revealed that “ferroptosis” and “TNF signaling pathway” were significantly enriched among the upregulated pathways. Other studies have found that ferroptosis (a lipid peroxidation-driven, iron-dependent form of cell death) and the activation of its pathways may be associated with elevated levels of MDA and cell membrane damage in gill tissue [32]. Based on this, we speculate that ferroptosis may be involved in the response to acute heat stress; however, this requires further in-depth investigation. In addition, activation of the TNF signaling pathway is typically associated with pro-inflammatory and pro-apoptotic cascades [33], which may explain the widespread distribution of TUNEL-positive cells in the gill tissue observed in this study. Conversely, downregulated genes were significantly enriched in pathways such as “steroid biosynthesis” and “GPI-anchored protein biosynthesis.” We speculate that the downregulation of gene expression involved in steroid synthesis (e.g., cortisol precursors) may be related to the body’s substantial consumption of steroid hormones in response to acute heat stress [34], although this requires further experimental confirmation.

Based on these findings, we have determined that acute heat stress may induce apoptosis by activating inflammatory responses and altering the expression of genes involved in pathways such as TNF signaling. However, this study has limitations that require further investigation; for example, it examined only acute heat stress at a single time point (12 h) and therefore cannot reflect the chronic adaptive mechanisms that may occur under slower heating rates or prolonged exposure in natural environments. Our study helps elucidate the immediate molecular and physiological responses to acute heat stress, but should not be directly extrapolated to long-term adaptation or evolutionary plasticity. Future research will include multiple time points, varying temperature gradients, and chronic stress experiments to gain a more comprehensive understanding of the adaptive potential of high-altitude fish to climate warming, thereby providing a solid molecular foundation for the conservation of *G. eckloni* and the breeding of heat-tolerant strains.

## 5. Conclusions

This study examined the multifaceted effects of acute heat stress on the gill tissue of *G. eckloni*. Histological and apoptosis analysis showed that acute heat stress significantly impaired gill tissue structure, as indicated by curled gill filament, edema, and vacuolar degeneration, and induced cell apoptosis. At the same time, acute heat stress induced significant stress responses and changes in oxidative stress markers, as shown by increased levels of total ATPase, total antioxidant capacity, glucose, and cortisol in serum, as well as upregulation of MDA, CAT activity, and LDH activity in gill tissue. Transcriptomic analysis identified 2304 DEGs, mainly involved in pathways related to inflammatory responses, TNF signaling, ferroptosis, and cell adhesion molecules, while gene expression was downregulated in pathways associated with steroid biosynthesis and PPAR signaling. In summary, we hypothesize that acute heat stress causes structural damage to the gill tissue of G. eckloni by affecting inflammation- and apoptosis-related pathways and disrupting redox balance.

## Figures and Tables

**Table 1:** Results of preprocessing for sequencing data quality.

**Sample**	**Raw Reads (M)**	**Raw Bases (G)**	**Clean Reads (M)**	**Clean Bases (G)**	**Valid Bases (%)**	**Q30 (%)**	**GC (%)**
AH 1	47.58	6.85	44.65	6.43	93.83	94.34	47.11
AH 2	46.41	6.65	43.17	6.18	93.01	93.98	46.66
AH 3	43.83	6.3	41.08	5.91	93.72	94.3	46.8
CG 1	47.45	6.85	44.66	6.45	94.11	94.54	46.44
CG 2	49.83	7.2	47.03	6.8	94.38	94.42	46.48
CG 3	41.9	6.05	39.43	5.69	94.11	94.27	46.61

**Table 2:** Details of the 10 genes selected for validation.

**Comparison Group**	**Pathways**	**Gene Annotated**	**Abbreviation**	**Up/Down**
AH vs. CG	Serotonergic synapse	Sodium-dependent serotonin transporter	*Slc6a4*	Up
	TNF signaling pathway	Cyclic AMP-dependent transcription factor ATF-7	*Atf7*	Up
	Motor proteins	Troponin I, fast skeletal muscle	*Tnni2*	Up
		Kinesin-like protein KIF13A	*Kif13a*	Down
	PI3K-Akt signaling pathway	DNA damage-inducible transcript 4 protein	*Ddit4*	Up
		Retinoic acid receptor RXR-alpha-A	*Rxraa*	Down
	Wnt signaling pathway	Dickkopf-related protein 2	*Dkk2*	Up
		Frizzled-2	*fzd2*	Down
	FoxO signaling pathway	Serine/threonine-protein kinase PLK4	*Plk4*	Down
	MAPK signaling pathway	Voltage-dependent calcium channel gamma-6 subunit	*Cacng6*	Down

## Data Availability

The original contributions presented in this study are included in the article/Supplementary Materials. Further inquiries can be directed to the corresponding author.

## Supplementary Materials

The following supporting information can be downloaded at: https://www.mdpi.com/article/10.3390/ani16121762/s1. Figure S1: Analysis of expression levels of protein-coding genes. (A) Bar chart showing the number of identified genes; (B) Box-and-whisker plot of gene expression levels; (C) Correlation coefficient plot between samples; (D) Results of cluster analysis between samples. Figure S2: Analysis results of the top 30 genes identified by GO enrichment analysis of differentially expressed genes. Figure S3: Information from the top 20 bubble plots of the KEGG enrichment analysis. Table S1: Results of the read alignment rate statistics against the reference genome. Table S2: Detailed information on differentially expressed genes between the two groups. Table S3: Details of the GO term enrichment analysis. Table S4: Information on the KEGG pathway analysis results for the gene. Table S5: Detailed information about the primers used in real-time quantitative PCR.

## Abbreviations

The following abbreviations are used in this manuscript:

MDA	Malondialdehyde
SOD	Superoxide dismutase
CAT	Catalase
LDH	Lactate dehydrogenase
T-AOC	Total antioxidant capacity
Glu	Glucose
DEGs	Differentially expressed genes
ROS	Reactive oxygen species
