# Supplementary figures and images for: Multi-Level Effects of Acute Heat Stress on Gill Tissue of Gymnocypris eckloni: Integrating Histopathology, Biochemistry, Apoptosis and Transcriptomics
Source: Animals (Basel). 2026 Jun 8;16(12):1762. doi: 10.3390/ani16121762 (PMC13296344; doi:10.3390/ani16121762)

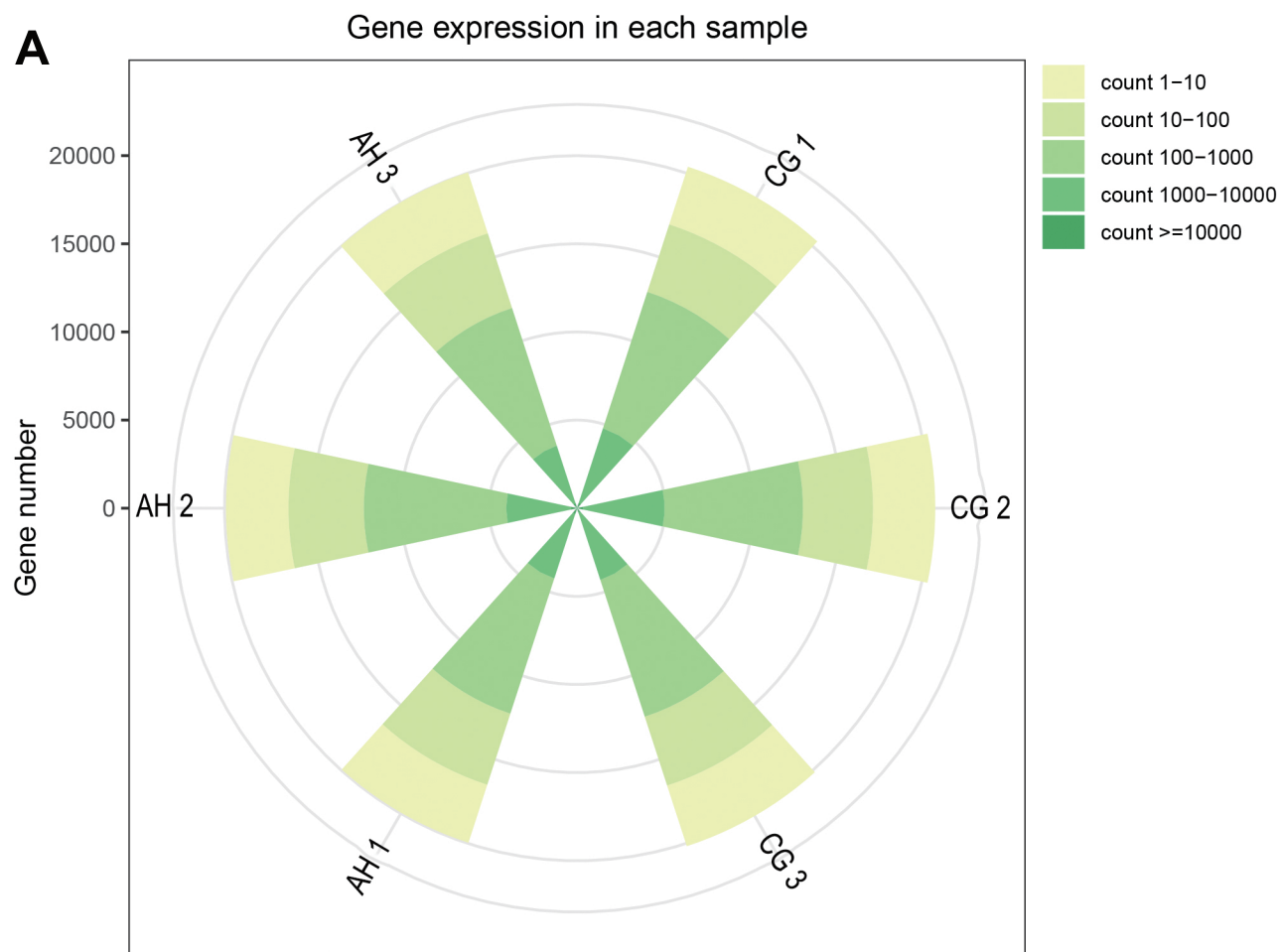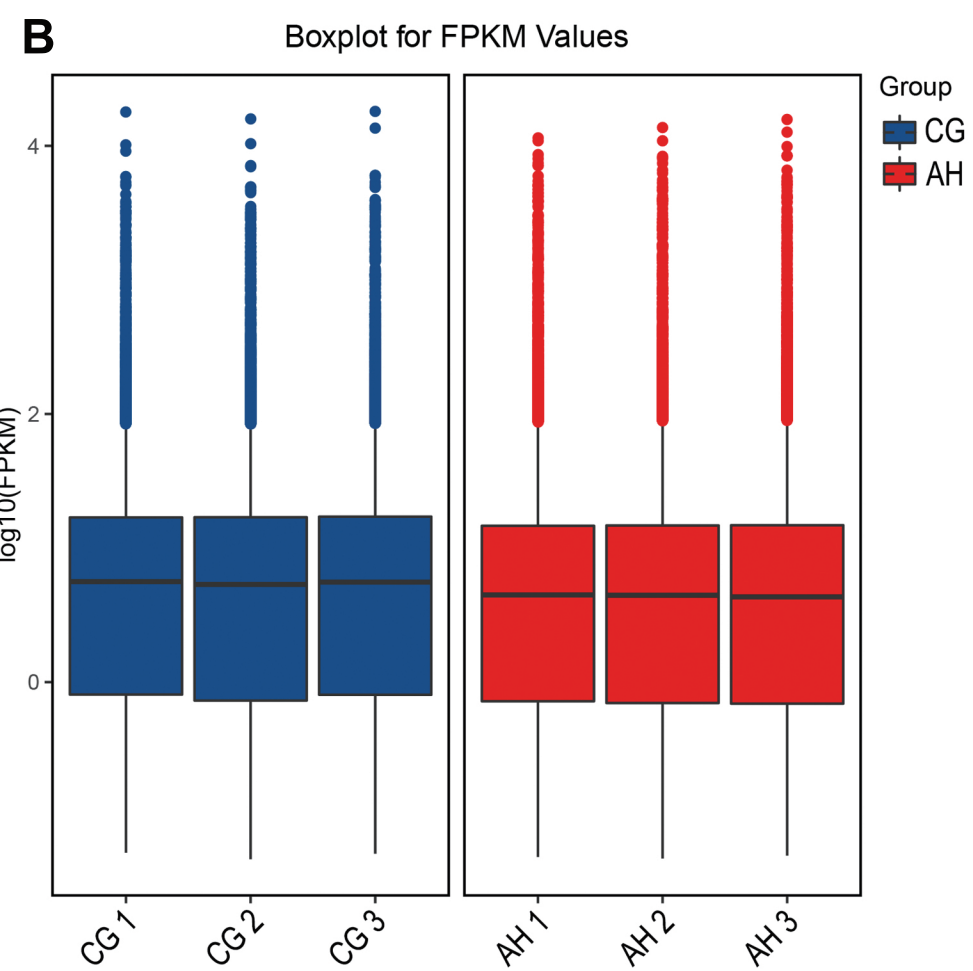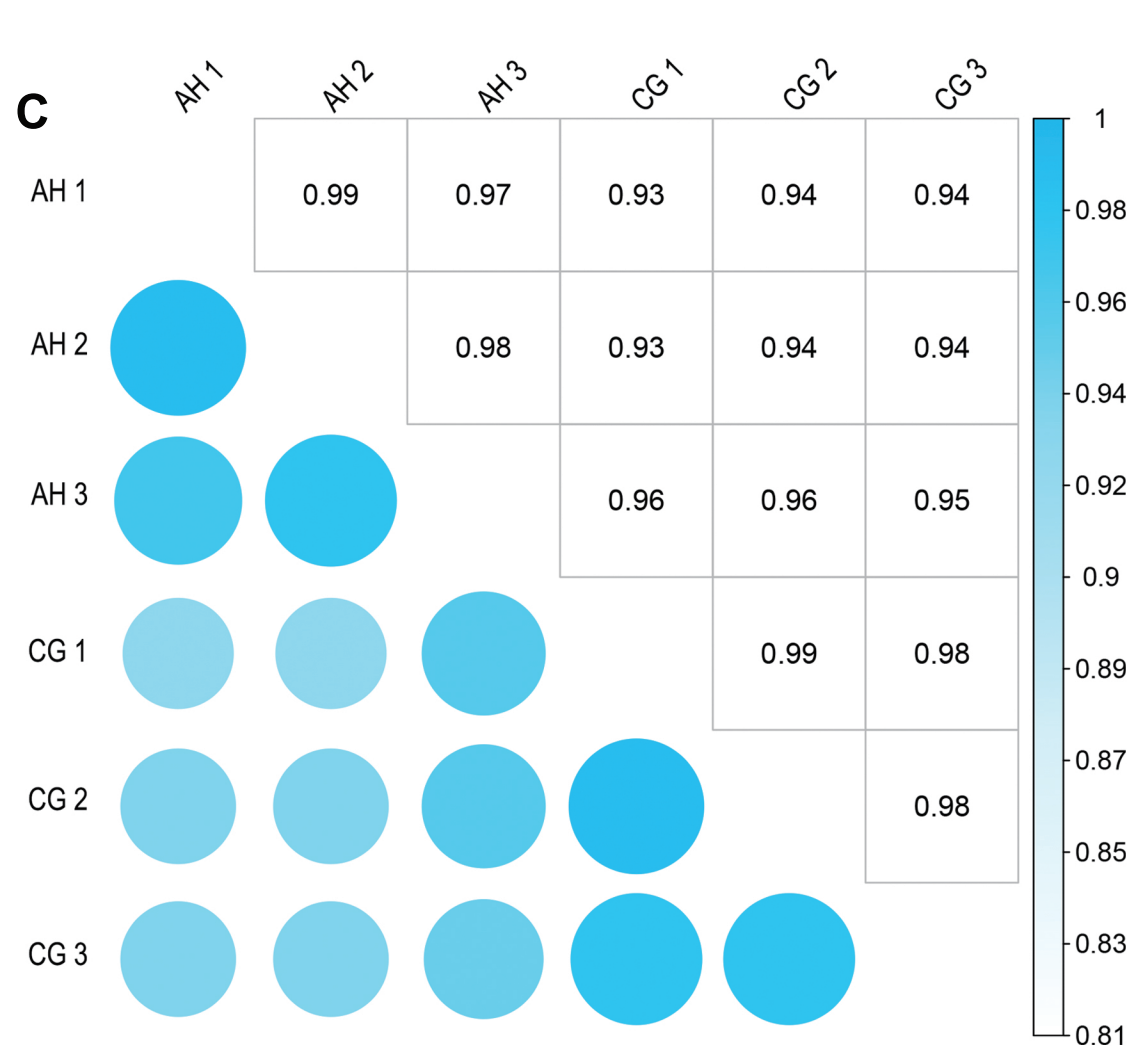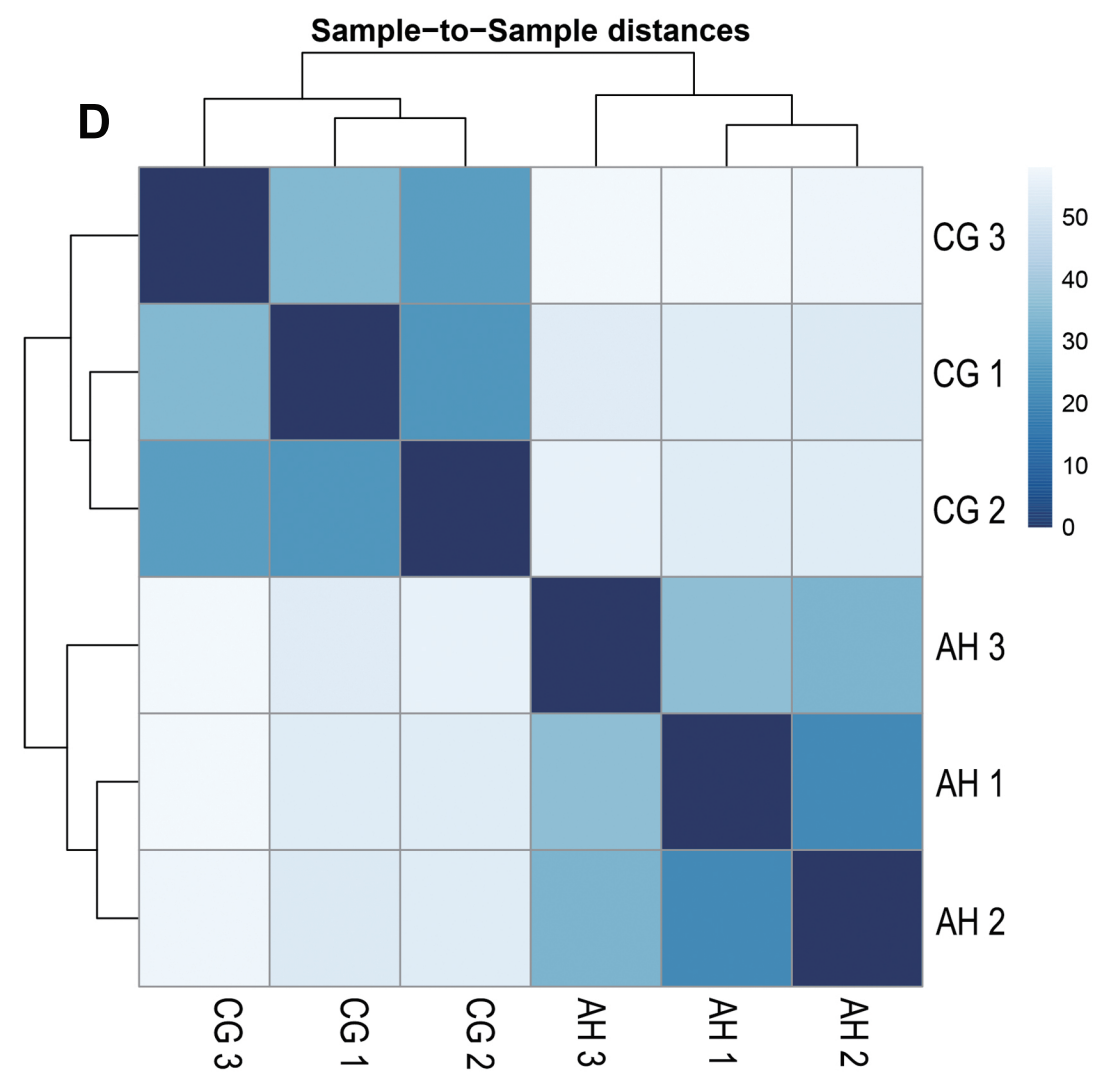

Supplement: Supplementary file 1 [file animals-16-01762-s001.zip › Supplementarty figures/Figure S1.pdf]

# AH-vs-CG(Total) Top 30 GO Term

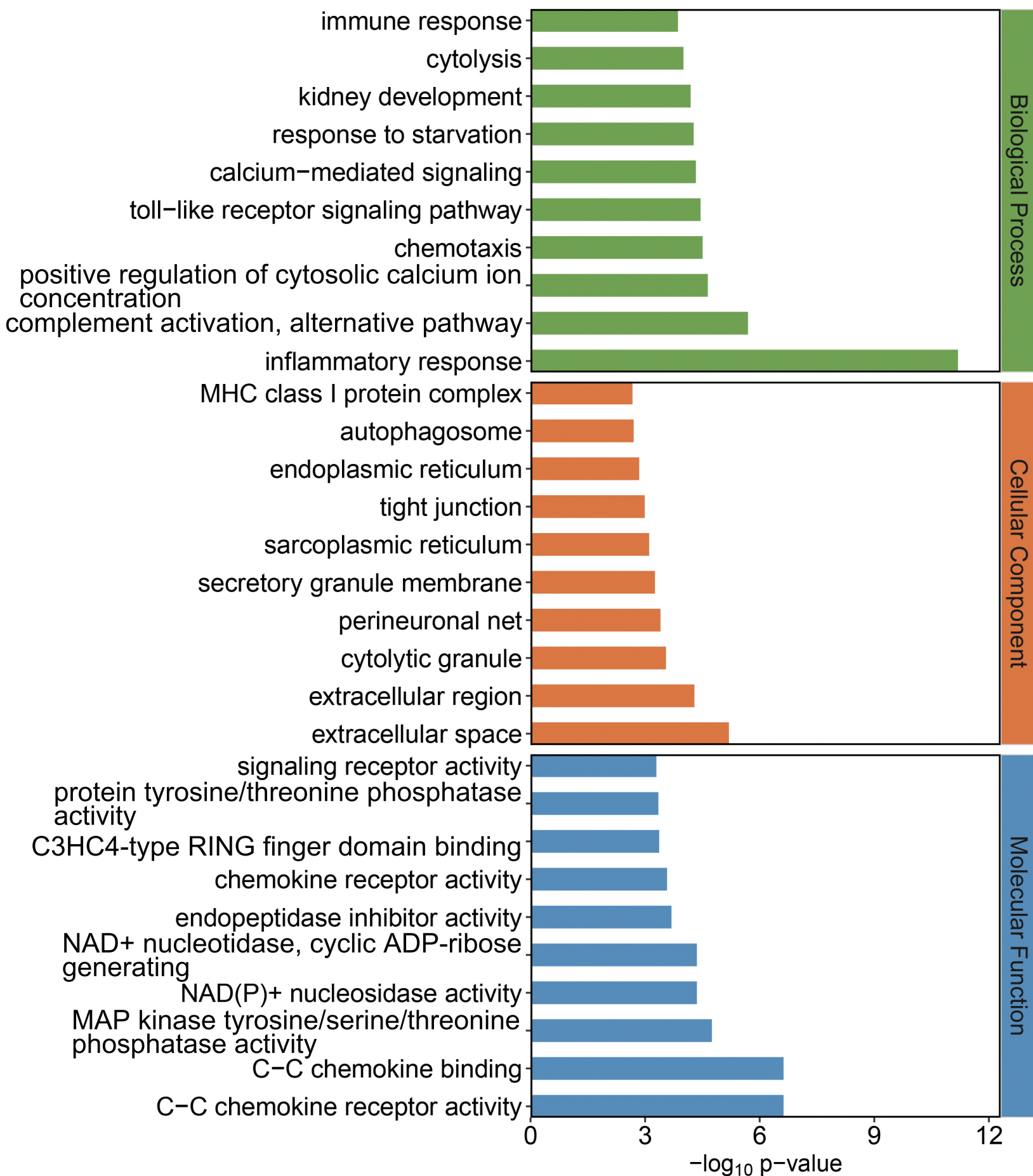

Supplement: Supplementary file 1 [file animals-16-01762-s001.zip › Supplementarty figures/Figure S2.pdf]

# AH-vs-CG(Total) KEGG Enrichment top 20

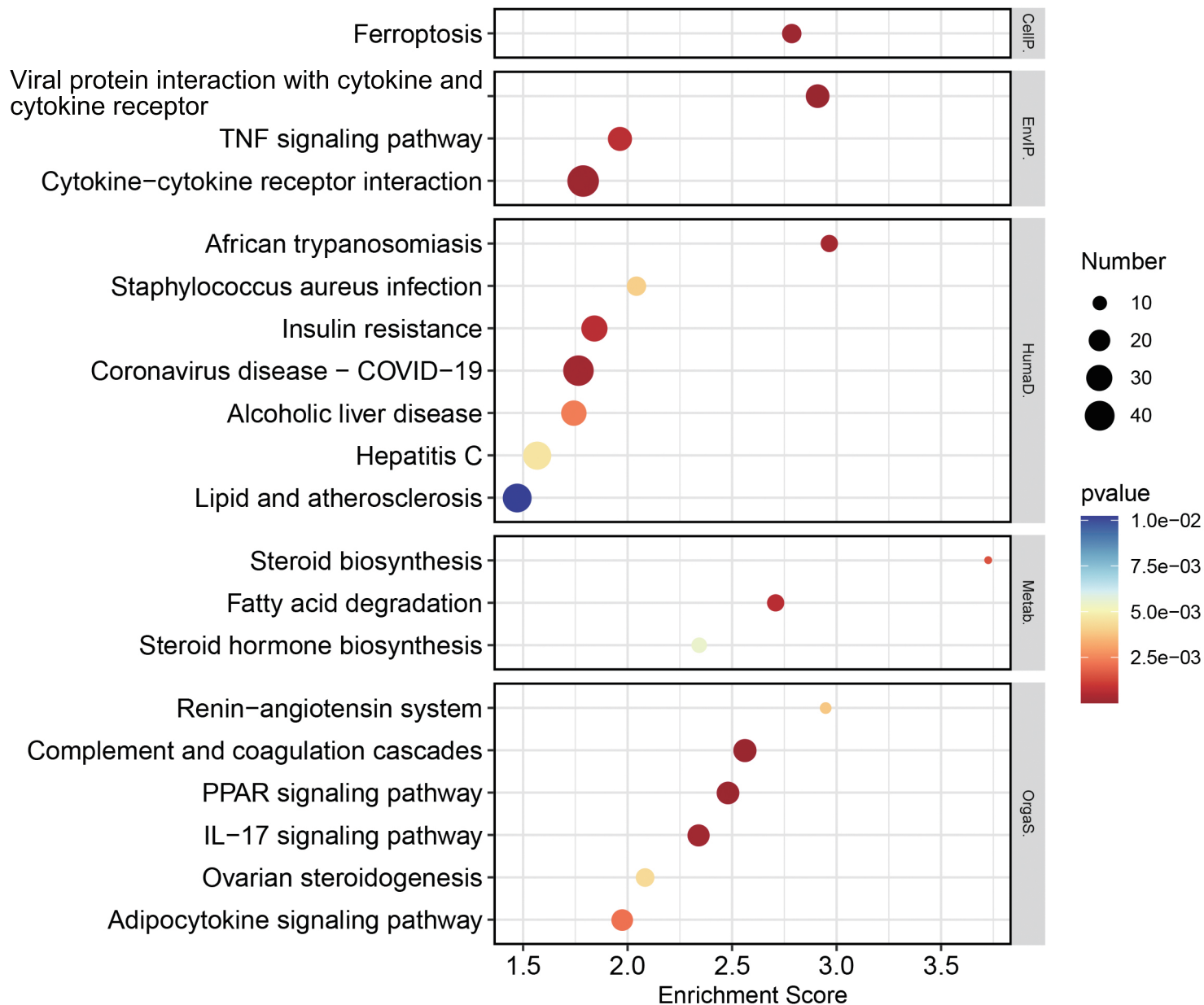

Supplement: Supplementary file 1 [file animals-16-01762-s001.zip › Supplementarty figures/Figure S3.pdf]
